# Cost Analysis of Noninvasive Helmet Ventilation Compared with Use of Noninvasive Face Mask in ARDS

**DOI:** 10.1155/2018/6518572

**Published:** 2018-02-08

**Authors:** Kwadwo Kyeremanteng, Louis-Philippe Gagnon, Raphaëlle Robidoux, Kednapa Thavorn, Dipayan Chaudhuri, Daniel Kobewka, John P. Kress

**Affiliations:** ^1^Department of Medicine, Division of Critical Care, University of Ottawa, Ottawa, ON, Canada; ^2^Department of Critical Care Medicine, Queen's University, Kingston, ON, Canada; ^3^University of Ottawa, Ottawa, ON, Canada; ^4^Clinical Epidemiology Program, The Ottawa Hospital Research Institute, The Ottawa Hospital, Ottawa, ON, Canada; ^5^School of Epidemiology, Public Health and Preventive Medicine, University of Ottawa, Ottawa, ON, Canada; ^6^Institute of Clinical and Evaluative Sciences (ICES uOttawa), Ottawa, ON, Canada; ^7^Department of Medicine, Division of General Internal Medicine, University of Ottawa, Ottawa, ON, Canada; ^8^University of Chicago Medicine, Chicago, IL, USA

## Abstract

Intensive care unit (ICU) costs have doubled since 2000, totalling 108 billion dollars per year. Acute respiratory distress syndrome (ARDS) has a prevalence of 10.4% and a 28-day mortality of 34.8%. Noninvasive ventilation (NIV) is used in up to 30% of cases. A recent randomized controlled trial by Patel et al. (2016) showed lower intubation rates and 90-day mortality when comparing helmet to face mask NIV in ARDS. The population in the Patel et al. trial was used for cost analysis in this study. Projections of cost savings showed a decrease in ICU costs by $2527 and hospital costs by $3103 per patient, along with a 43.3% absolute reduction in intubation rates. Sensitivity analysis showed consistent cost reductions. Projected annual cost savings, assuming the current prevalence of ARDS, were $237538 in ICU costs and $291682 in hospital costs. At a national level, using yearly incidence of ARDS cases in American ICUs, this represents $449 million in savings. Helmet NIV, compared to face mask NIV, in nonintubated patients with ARDS, reduces ICU and hospital direct-variable costs along with intubation rates, LOS, and mortality. A large-scale cost-effectiveness analysis is needed to validate the findings.

## 1. Introduction

Our population is aging, hospital admissions are getting more frequent with a longer length of stay (LOS), intensive care and hospital occupancy rates are climbing, and healthcare-associated expenditures are increasing [1, 2]. Intensive care costs alone totalled 108 billion US dollars in 2010, nearly double that of 2000 (56 billion US dollars). ICU costs account for 13.2% of hospital expenditures and 0.72% of the gross domestic product in the United States, a 32% percent rise from 2000 to 2010 [1, 2]. It is estimated that a single day in the intensive care unit (ICU) costs 2500–4300 US dollars per patient, representing a 61.1% increase in costs over the same time period with the use of new medications, technologies, and specialized care [1–4]. Moving forward, finding ways to reduce ICU costs will reduce the financial burden of increasing utilization of ICU care. New practices focusing on cost-effectiveness will be key by evaluating the effectiveness of the practice on patient outcomes as well as the resources required to implement it [5].

Acute respiratory distress syndrome (ARDS) has a worldwide prevalence of 10.4% in ICUs and an overall 28-day mortality of 34.8% [6]. In the absence of a proven mortality benefit, noninvasive ventilation (NIV) is used in up to 30% of patients with ARDS [6–8]. In June 2016, Patel et al. published the first randomized controlled trial comparing helmet and face mask NIV for patients with ARDS [7]. The patients who received helmet NIV had a reduction in intubation rates, ICU LOS, and 90-day mortality, as well as increased ventilator-free days. Intubation rates were 61.5% in the face mask group, as opposed to 18.2% in the helmet group, giving an absolute risk reduction of 43.3% (95% CI 24.3–62.4%, *p* < 0.001). In fact, the trial was stopped early for efficacy at the first interim analysis after one-third of planned patient enrollment.

Increased ventilator days are associated with longer LOS, higher risk of complications of intubation in ARDS such as pneumonia [9], delirium [10], and ICU-acquired weakness [11], and a higher risk of mortality. Reducing intubation rates is therefore expected to reduce the number of complications and have significant cost savings.

Helmet NIV has been shown to provide similar oxygenation when compared to face mask NIV while providing better patient tolerability, less air leaks, and a universal size independent of facial anatomy [12, 13].

This study was conducted to quantify the potential ICU and hospital cost savings of helmet NIV compared to face mask NIV in ARDS.

## 2. Methods

The population used to calculate ICU and hospital costs consists of patients with ARDS, treated with face mask or helmet NIV, studied in the randomized controlled trial by Patel et al. [7]. The total and individual patient costs of both study groups were calculated based on their reported ICU and hospital LOS. A cost analysis model proposed by Lord et al. [14] based on cost values published by Kahn et al. [15] was used to estimate average daily cost, which was then multiplied by the LOS values. This model calculates daily direct-variable costs in the ICU and on hospital wards. It assumes that ICU days are more expensive than ward days, that all ward days have the same cost, that the first ICU day is the most expensive, and that the ICU cost decreases daily until day 5. All costs were inflated to 2016 US dollars using the overall consumer price index reported by the Bureau of Labor Statistics [16]. We calculated the difference in costs by subtracting the costs of the helmet NIV group from the face mask NIV group. 95% confidence intervals were estimated using bootstrap resampling procedures with 1000 iterations. Potential cost savings were projected by assuming a 10% prevalence of ARDS, 30% use of NIV in ARDS, and identical cost of purchase of helmet and face mask NIV to then multiply average cost savings per patient with the potential number of ARDS patients per year treated with NIV.

## 3. Results

For the purpose of this paper, the population examined by Patel et al. was used as the study sample (as detailed in [7]). The total number of patients is 83, 39 of which were treated with face mask NIV, and 44 with helmet NIV. Both groups were comparable in terms of age, severity of illness, medical history, reason for acute respiratory failure, PaO_2_  :  FiO_2_ ratio, and the amount of respiratory support on NIV. Outcomes are presented in Table 1. There was a statistically significant reduction in endotracheal intubation (−43.3%, *p* < 0.001) and increase in ventilator-free days (8.4, *p* < 0.001) with helmet NIV. There was also a reduction in ICU LOS, hospital LOS, and 90-day mortality with helmet NIV.

Absolute differences in cost are shown in Table 2. With a significant reduction in ICU and hospital LOS in the helmet NIV group, associated ICU and hospital costs were reduced by 2527 US dollars and 3103 US dollars per patient, respectively. The total cost saving in the care of the helmet NIV group was 71842 US dollars.

A sensitivity analysis was conducted to assess potential variations in ICU and hospital costs, according to varying ICU lengths of stay, for either the face mask or the helmet group, or both. As shown in Table 3, even by increasing the helmet group's ICU LOS, helmet NIV consistently represented a cost-saving method to treat patients with ARDS. For example, a one-day increase in ICU LOS for the helmet group and a steady ICU LOS for the face mask group lead to a reduction in ICU and hospital costs of 1712 US dollars and 2288 US dollars per patient, respectively, favoring helmet NIV. To note, these analyses manipulated LOS regardless of outcome, which was expected to remain stable across groups throughout the sensitivity analysis. However, as helmet NIV group patients are expected to show a greater proportion of successful outcomes, the sensitivity analysis may be seen as conservative in the magnitude of the advantage it indicates for helmet use.

## 4. Discussion

This cost analysis shows a consistent reduction in cost associated with the use of helmet NIV in the ARDS population studied by Patel et al. [7] along with a reduction in intubation rates, LOS, and mortality. Potential savings could be illustrated despite variations in LOS with the sensitivity analysis, and these savings were extrapolated to differing volumes of ARDS. This analysis represents an important step in finding a cost-efficient, well-tolerated, and easily applicable way to deliver care to patients with ARDS who are not intubated at the time of assessment and admission to the ICU. As mentioned, this represents up to 30% of patients with ARDS. Importantly, single interventions with a mortality benefit in the care of ICU patients such as this one are uncommon [17].

The magnitude of potential savings at a national level is immense. 5.7 million patients are admitted to an ICU each year in the United States [18], and 10.4% of them are assumed to have ARDS [6]. This corresponds to 592800 patients who are admitted with ARDS yearly. Assuming that up to 30% of these patients are treated with NIV, which is to say 177840 patients, the use of the helmet interface would lead to potential total savings of 449 million US dollars when compared to the face mask interface.

Limitations to this cost analysis lie in the assumptions of the model. Firstly, calculations were based on a randomized and controlled, but single-center, unblended, and small study population. This center was also accustomed to using the helmet NIV interface. In order to avoid unwarranted variability, centers unaccustomed to the use of helmet NIV would need to be included in future studies, to avoid selection bias based on established experience with the interface. Secondly, costs were obtained from a model developed from a separate single-center's financial data. In order to establish that helmet NIV is a truly cost-effective way of treating nonintubated patients with ARDS, a large multicenter health economic evaluation, comparing costs and outcomes of the helmet to the face mask NIV, interfaces would need to be done. Hospitals would track direct costs concurrently and include the cost of the apparatus of NIV (face masks, helmets, etc.) in the analyses, in order to provide exact rather than projected costs. It would not be possible to perform a blinded study as patients and healthcare providers would evidently notice with which group of NIV delivery they were interacting. However, the bias of not being blinded is unlikely to affect results due to the presence of objective indications for intubation and progression of respiratory illness. Also this study's costing methods do not incorporate major cost drivers such as extracorporeal membrane oxygenation (ECMO) or renal replacement therapy, which may be seen in severe cases of ARDS.

It is noteworthy that the majority of costs in the ICU are fixed. An intervention such as the use of helmet NIV in ARDS has the potential to decrease direct-variable ICU costs, which usually account for 20% of yearly ICU expenses. Savings in direct-variable costs would create opportunities to fund other initiatives such as early mobility, which has been shown to increase ventilator-free days and functional outcomes as well as decreasing LOS [19–22].

## 5. Conclusion

The use of the helmet NIV interface, when compared to the face mask interface in the management of nonintubated patients with ARDS, reduces intubation rates and mortality, as well as ICU and hospital costs. The savings from such an intervention are potentially immense from a national standpoint, and they represent an opportunity to redistribute direct-variable ICU funds to other evidence-based beneficial therapies, such as early mobility initiatives.

Future studies could conduct a cost-effectiveness analysis comparing cost and health outcomes associated with each NIV interface. Cost-effectiveness analyses are crucial to minimize preventable expenses and ensure sustainability in the health sector, an ever-growing need in today's climate. These analyses could be incorporated in large-scale trials looking at new interventions and clinical guideline development by focusing on cost effectiveness. They could also be integrated in the comparison of two or more therapies with already established benefits for the same disease or syndrome. A relevant example is that of the comparison of outcomes and costs associated with the use of helmet NIV and high-flow nasal oxygen in acute hypoxic respiratory failure. Those potential studies would enrich this domain of research even further and contribute to build a more sustainable healthcare system for aging populations.

## Figures and Tables

**Table 1:** Outcomes and adverse events in the Patel et al. [7] study.

	Face mask (*n* = 39)	Helmet (*n* = 44)	Absolute difference (95% CI)	*p* value
Primary outcomes, *n* (%)				
Endotracheal intubation	24 (61.5)	8 (18.2)	−43.3 (−62.4 to −24.3)	<0.001
Reason for intubation				
Respiratory failure	20 (83.3)	3 (37.5)	−45.3 (−82.5 to −9.1)	0.01
Circulatory failure	3 (12.5)	0 (0)	−12.5 (−25.7 to 0.7)	0.55
Neurologic failure	1 (4.2)	5 (62.5)	58.3 (24.8 to 92.8)	0.001
Secondary outcomes, median (IQR), days				
Ventilator-free days	12.5 (0.49 to 28)	28 (13.7 to 28)	8.4 (13.4 to 3.4)	<0.001
ICU LOS	7.8 (3.9 to 13.8)	4.7 (2.5 to 8.7)	−2.76 (−6.07 to 0.54)	0.04
Hospital LOS	15.2 (7.8 to 19.7)	10.1 (6.5 to 15.9)	−2.92 (−8.47 to 2.63)	0.16
Mortality, *n* (%)				
Hospital	19 (48.7)	12 (27.3)	−21.4 (−41.9 to −1.0)	0.04
90 days	22 (56.4)	15 (34.1)	−22.3 (−43.3 to −1.4)	0.02
Adverse events				
Mask deflation	0 (0)	2 (4.5)		
Skin ulceration	3 (7.6)	3 (6.8)		

**Table 2:** Cost analysis of ARDS population treated with NIV in the Patel et al. [7] study (95% CI) per patient.

	Face mask (*n* = 39)	Helmet (*n* = 44)	Absolute difference (95% CI)
ICU LOS (days)	7.8 (3.9–13.8)	4.7 (2.5–8.7)	—
ICU cost (US dollars)	10773	8246	2527 (2251–2817)
Hospital LOS (days)	15.2 (7.8–19.7)	10.1 (6.5–15.9)	—
Hospital cost (US dollars)	12938	9835	3103 (2829–3392)
Total direct-variable cost for cohort (US dollars)	504582	432740	71842 (69895–73740)

**Table 3:** Sensitivity analysis assuming variability in ICU LOS in different NIV study groups.

		ICU costs (US dollars)			Hospital costs (US dollars)		
		Face mask	Helmet	Absolute difference	Face mask	Helmet	Absolute difference
Change in ICU LOS (days)	Affected study population						
+1	Face mask	11588	8246	3342	13765	9835	3930
	Helmet	10773	9061	1712	12938	10650	2288
	Face mask and helmet	11588	9061	2527	13765	10650	3115
−1	Face mask	9958	8246	1712	12135	9835	2300
	Helmet	10773	7380	3393	12938	8968	3970
	Face mask and helmet	9958	7380	2578	12135	8968	3167
